# Evaluation of the star family doctors training program: an observational cohort study of a novel continuing medical education program for general practitioners within a compact medical consortium: a quantitative analysis

**DOI:** 10.1186/s12909-023-04210-7

**Published:** 2023-04-17

**Authors:** Ling-Bo Liang, Xu Li, Xiang-Ping Liu, Cai-Zheng Li, Dan Luo, Feng Liu, Ting-Rui Mao, Qiao-Li Su

**Affiliations:** 1 General Practice Ward/International Medical Center Ward, General Practice Medical Center, West China Hospital, Sichuan University, Chengdu, 610041 China; 2 Department of Primary Health Care, Health Commission of Sichuan Province, Chengdu, 610041 China; 3 Department of Primary Health Care, The fourth People’s hospital of Dazhu County, Dazhou, 635100 China

**Keywords:** Continuing professional development, General practice, Program evaluation, Primary care

## Abstract

**Introduction:**

To determine the effectiveness of the Star Family Doctors Training Program, a comprehensive Continuing professional development (CPD) program for general practitioners (GPs) in a compact medical consortium.

**Patients and Methods:**

Observational cohort study with a quantitative analyses in primary health care institutions in Sichuan Province. The interventions were as following: (1) The Star Family Doctors Training Program is a full-time, local government allocation program certified by the Health Department of Sichuan Province, emphasizing small group learning and practice, and using standard patients and medical patient simulators; 30 participants were selected by their institutions. (2) The control group underwent a self-financed after-work CPD program using conventional lectures; 50 participants were self-selected. Short-term effectiveness assessed using immediate post-training tests and self-evaluations; long-term (1 year) effectiveness evaluated using self-reported surveys.

**Results:**

The study involved 80 GPs (28.75% men; mean age: 38.2 ± 9.2 years). The average post-training total score was higher in the STAR group than in the control group (72.83 ± 5.73 vs. 68.18 ± 7.64; *p* = 0.005). Compared to the controls, STAR participants reported seeing more patients (all *p* < 0.05), and had more patients who signed family-doctor contracts (*p* = 0.001) as well as increased patient satisfaction (*p* = 0.03), respectively. STAR-group trainees appraised the program higher and were more willing to recommend it to colleagues (90% vs. 64%, *p* = 0.011).

**Conclusion:**

The Star Family Doctors Training Program achieved good responses and provides a reference for future CPD programs.

**Supplementary Information:**

The online version contains supplementary material available at 10.1186/s12909-023-04210-7.

## Introduction

Continued professional development (CPD) is required for updated skills and knowledge. Among CPD, continuing medical education (CME) plays an important role in improving doctors’ professional knowledge and skills, optimizing medical behavior, and promoting medical safety [1–2]. Levine has stated that the main purpose of CME is to “maintain and improve clinical performance”.[3–4] Other authors believe that “Only when the acquired knowledge actually offers an opportunity for changes in practice, it is meaningful”.[5–6] CME is aimed at working doctors and nurses who have a certain amount of clinical experience. Adults have very different learning needs and learning styles from children and adolescents: adults are results oriented, autonomous, self-directed, relevancy oriented, and practical, and their past experience may promote or interfere with the training. [7–10] Therefore, effective CME should emphasize practicality and actionability, attach importance to self-evaluation, closely integrate with clinical practice, and protect participants from the interference caused by daily work and the cost of food and accommodation during the training.

Serving as the first contact and “gatekeeper” of the public, the primary health care (PHC) system plays an indispensable role in providing preventive and basic medical care to the entire population. It is generally accepted that high-quality primary care should involve continuity, comprehensiveness, coordination, and patient orientation. [11] Although China has made remarkable efforts to strengthen the quality of its PHC during the past decade,[12–13] two recent reviews published in the *Lancet* have pointed out that several inadequacies still exist in the PHC system in China, [11, 14] for example, the low quality of the diagnostic process and outcomes, the underperformance of PHC with respect to the management of noncommunicable chronic diseases, substantial gaps in the education of doctors in the PHC system, and widespread low job satisfaction and high occupational burnout. These inadequacies act as obstacles to the Healthy China 2030 blueprint, a national public health strategy; [15] thus, there is an urgent need for both the government and institutions to address the above shortcomings.

It can be seen from the above that to promote the PHC quality in China, it is very important to design and continuously and extensively promote a CME training program that is closely integrated with the community, is strongly practical, improves the professional quality and ability of grassroots medical workers, and promotes their cooperation with higher-level hospitals. [16–19] By referring to the available literature on existing CME projects, we believe that a future training program should have the following characteristics: (1) local financial and special funding support, and a health administrative department that coordinates the work of medical institutions, which can minimize the stress of doctors participating in the program in terms of leave and expenses; [20–21] (2) general medicine courses that are mainly implemented in hospitals affiliated to comprehensive universities with long-term cooperative relationships with grassroots medical institutions, that involve the joint participation of relevant specialties, and that set teaching objectives after taking into account self-evaluations and the needs of the medical system; [22–23] (3) a focus on practicality and maneuverability, assessments that are closely integrated with daily clinical work, and an emphasis on student-teacher interactions during the course of training, with the aim of improving not only the knowledge and skills of the participants but also their mode and concept of medical practice; [24] and (4) some type of certification for grassroots health care workers who successfully complete the training that is of positive significance to their career to encourage more general practitioners (GPs) to participate in the training. [11].

In recent years, many CME programs for primary care providers have been carried out around the world, but there are some deficiencies in the existing research. Most CME projects focus on training for a particular disease or use a single teaching method. [25–31] The main weakness of the few integrated training programs is the lack of control studies and cross-sectional studies, with only examination results serving as an evaluation indicator. [32] Therefore, our research group intended to work with a team of general medicine and specialist teachers in a general hospital to design a new type of CME course at two weeks of full-time centralized training for the Star Family Doctors Training Program (the STAR group) and on-weekends/off-job training lasts for 3 months of the conventional training program using decentralized methods for control group that covers (1) basic diagnosis and treatment methods and medical techniques based on the needs of the community, (2) the comprehensive management of common chronic noncommunicable diseases in the community, (3) the practice and management of family doctors, and (4) the training of community teachers. Specifically, the detail description of this CME course is shown below.

We designed a centralized CME course training program with teaching methods that included small group discussions, standardized patients, workshops, and traditional lectures. According to the Stufflebeam’s Context, Inputs, Processes, Products (CIPP) model, [33] the assessment methods included objectives, structure of training, measurement tools, implementation program, immediate evaluation, and long-term evaluation. The post-training test results and self-evaluations were taken as the immediate evaluation indices, and the participants’ practice of the training content and their improvement in medical quality at 1 year after the training were taken as the long-term evaluation indices. The results of this CME program were comparatively analyzed against those of a conventional training program using decentralized methods as above description. We hope that our study will provide a valuable reference for CME and training projects to meet the real needs of the community and improve the quality of grassroots health care.

## Patients and methods

### Study design

We conducted an observational cohort study based on the quantitative methods, from September 2019 to February 2021. The “Star Family Doctors Training Program” is part of the provincial CME program, and is administratively and financially supported by the Health Commission of Sichuan Province and Sichuan Provincial Finance Department. The whole training program involved collaboration of the Health Commission of Sichuan Province and the “West China Hospital and Chenghua Urban Area Medical Service Alliance”, a novel Compact Medical Consortium including the leadership group, West China Hospital departments, grassroots organizations, information platforms, and urban residents and health care teams, aiming to provide integrated, homogeneous, and accessible medical services centered on residents’ health. [34] The participants recruited for the STAR group were selected from their working institutions, and underwent two weeks of full-time centralized training. A control program involving conventional lecture learning was also designed; participants voluntarily applied for the control program, which was self-financed and held on weekends/off-job training lasts for 3 months. A detailed comparison of the STAR and control groups is shown in Table 1 and **Supplementary file**1.

**Table 1:** Comparison between the STAR group and the control group

Items	STAR group(n = 30)	Control group(n = 50)
The source of the trainees	Elected from their health care institutions	Voluntarily applied for the training program
Training expenses	Local government allocations	At one’s own expense
Training time	Two weeks of full-time centralized training	On-weekends/off-job training lasts for 3 months
*Training methods*		
Traditional lecture	√	√
Small group case study	√	×
Workshop	√	√
Using Standard Patient	√	×
Using Medical Patient Simulator	√	×
*Real-time outcome measurement*		
Attendance rate	√	√
Post-training self-evaluation	√	√
Written examination score	√	√
Physical examination score	√	√
Inquiry/consultation score	√	√
Presentation of a common chronic condition management	√	√
SOAP case score	√	√
*Certification/Award*		
Trainees with total score > 60	Offer a certificate of “The STAR family doctor” with the official seal of Health Commission of Sichuan Province	Offer a certificate of “The Compact Medical Consortium family doctor” with the official seal of West China Hospital
Trainees with total score > 80	(1) Offer a certificate of “The outstanding STAR family doctor” with the official seal of Health Commission of Sichuan Province, which will be issued on site of the Sichuan Provincial General Practice Annual Conference (2) Offer the authority of “Bidirectional referral medical service of General Practice Community Alliance of West China Hospital”, severe patients can be transferred to the department of general practice of West China Hospital through a particular referral mechanism.	Offer a certificate of “The outstanding Compact Medical Consortium family doctor” with the official seal of West China Hospital
*Twelve-months follow-up outcome measurement*		
Practical application of the training contents	√	√
Improvement in clinical performance	√	√
Salary changes after the training	√	√

*Abbreviations*: STAR = Star Family Doctors Training Program; SOAP = Subjective, Objective, Assessment, and Plan (Medica documentation format)

### Participants

The inclusion criteria were as follows: general practitioners (GPs) working in PHC institutions in Sichuan Province who (1) had a general practice qualification certificate, (2) had been working for over 3 years in total and over 1 year at their present health care institution in order to baseline consistence, and (3) were willing to attend and complete the entire training program as well as the 12-month follow-up. The exclusion criteria were participants working in solely administrative positions and those who had previously participated in a similar training course at any time.

### Participant recruitment

The official notice of the Star Family Doctors Training Program, as part of the provincial primary care quality promotion project, was issued by the Health Commission of Sichuan Province to all registered PHC institutions. Potential participants applied through their working institutions, and only one candidate was selected from each health care center. Eventually, 30 GPs were recruited in the STAR group. The control program, as one of the training courses of the General Practitioner Ability Enhancement Program of West China Hospital, was advertised through the official website of the hospital management office of West China Hospital. GPs in Sichuan Province voluntarily applied for the course through the website, and we finally enrolled 50 participants in the control group.

### Training program design

To better conform to the actual needs of the daily practice of PHC providers, the Star program was preceded by a training-requirements survey that was previously implemented by our research team in several PHC centers in Chengdu, the provincial capital city of Sichuan. [35] Based on the questionnaire results, the training program of the STAR group was designed as an integrated course containing 5 parts: (1) fundamental GP skills, (2) fundamental conceptions of general practice, (3) requisite skills for daily clinical practice in the community, (4) requisite abilities for daily clinical practice in the community, and (5) holistic common chronic-condition management. Multiple teaching methods were applied during different stages of the course. The details of the training program design are summarized in Table 2 for STAR group and **Supplementary file**1 for control group. The contents of the training program in the control group were identical to those of the training program in the STAR group; the only differences were in the teaching methods and in the program implementation (Table 1 and **Supplementary file**1).

**Table 2:** Teaching contents and methods of the STAR group

Items	Courses	Methods	Time allocation
*Fundamental GP clinical skills*	Consultation skills and doctor-patient communication	Standard Patient^†^	2 h
	SOAP medical record writing	Within-group practice	1 h
	Physical examination: including (1) Head and Neck; (2) Chest; (3) Abdomen; (4) Extremities.	Standard Patient demonstration and within-group practice	2.5 h for each part
*Fundamental conceptions of general practice*	(1) Clinical thinking of general practitioners (2) Whole-person health management experience sharing (3) Design and implementation of the contracted health service package (4) Operational management of the health management team	Lectures and case sharing	2 h 2 h 2 h 2 h
*Requisite skills for daily clinical practice in the community*	(1) Portable pulmonary function devices (2) Ambulatory blood pressure monitor (3) Dynamic blood glucose monitor (4) Patient education	Lectures, demonstration, and workshop practice	2.5 h 2.5 h 2.5 h 2 h
*Requisite abilities for daily clinical practice in the community*	(1) Cardio-pulmonary resuscitation and the Heimlich maneuvere (2) Thoracentesis, abdominocentesis, debridement, wound treatment (3) Common Electrocardiogram abnormality identification (4) Common chest imaging abnormality identification (5) Common Color Doppler ultrasound abnormality identification (6) Common laboratory tests abnormality identification	Lectures, demonstration, personal practice, and image/ECG reading practice	3 h 3 h 2.5 h 2.5 h 2.5 h 2.5 h
*Holistic common chronic condition management*	(1) Hypertension; (2) Diabetes mellitus; (3) Dyslipidemia and atherosclerosis; (4) Chronic pulmonary obstructive disease; (5) Hyperhyperuricemia and gout; (6) coronary heart disease; (7) Chronic heart failure; (8) Ischemic stroke and intracerebral hemorrhage; (9) Osteoporosis; (10) Prostatomegaly; (11) Chronic hepatitis B infection; (12) Hospital infection prevention.	Lectures, case sharing and within group discussion	3 h for each session^‡^

^†^ Standard Patient was invited from West China Hospital, Sichuan University

^‡^ The session of chronic hepatitis B infection for 2 h; the session of hospital infection prevention for 1 h

### Immediate post-training objective evaluation

The immediate post-training evaluation was designed as a mixed assessment (Table 3). The attendance of every trainee was recorded during each session, and only those who had an attendance rate higher than 90% and finished the qualitative self-evaluation were eligible to undergo the post-training evaluation, which included written examination, performance of outpatient service, and management of chronic conditions. The written examination, physical examination, and inquiry assessment were arranged soon after the training. SOAP medical records were requested to be submitted within 1 week after the training program, and the assessment of the presentation of real-world chronic-condition management was arranged 2 weeks after the training.

**Table 3:** Summary of the post-training tests *Abbreviations*: SOAP = Subjective, Objective, Assessment, and Plan (Medica documentation format)

Items	Test contents	Proportion of total score	Assessment setting
*Written examination*	choice questions, case analysis, ECG, radiological, and ultrasound image reading	30%	Two trainers went over and score the exam papers collectively
*The performance of outpatient service*			
Physical examination assessment	The assessment containing four parts: Head and Neck, Chest, Abdomen, extremities.	15%	Each trainee chose one part of the assessment by drawing lots, the performance was assessed by two trainers independently, and scored collectively
Inquiry assessment	the assessment was emphasized on five aspects: history taking, diagnosis and differential diagnosis, comprehensive evaluation, patient education and communication ability	15%	One trainer acted as the “patient” based on the scripts, the performance of the trainee was assessed by two other trainers independently, and scored collectively
*The management of chronic conditions*			
SOAP medical record writing	Each trainee was asked to hand in ten SOAP cases based on his/her practical clinic work, there were no restrictions of the choice of diseases	10%	Two trainers score the cases collectively
Presentation of chronic conditions management	Each trainee was asked to display one case of chronic condition management in 15 min based on his/her practical clinic work, there were no restrictions of the choice of diseases. The assessment was emphasized on five aspects: standardization, feasibility, clarity, confidence, and humanity	30%	The performance was assessed by five trainers independently, and scored collectively

*Abbreviations*: SOAP = Subjective, Objective, Assessment, and Plan (Medica documentation format)

### Immediate post-training self-evaluation

All self-evaluation items of the training effects were designed based on a 5-point Likert scale. The investigation contents included (1) enhancement of clinical ability via the training, (2) appropriateness of the training for the practical needs of the community, (3) accuracy and key points of the training, (4) assistance in standardization of diagnosis and treatment behavior, (5) improvement in teaching ability, and (6) inclination to recommend colleagues to participate in serial training. All items were rated on a 5-point scale from “disagree strongly” to “agree strongly” (scores: 1–5, respectively). In addition, the opinions and advice of all the trainees on both the training courses and test methods were collected.

### The 12-month post-training follow-up

All trainees in both the STAR group and the control group were invited to participate in our remote online academic communication group after the training program to share their clinical experience, discuss complicated cases, cooperate in hierarchical management, and broaden their horizons. Thus, we gradually built a relatively close connection with each trainee. An online questionnaire combined with personal interviews via telephone and text messages was conducted 1 year after the training had ended. The self-reported items included the practical application of the training contents, improvement in clinical performance, which represented as changes in the number of outpatients, patients who signed a family-doctor contract, patient satisfaction according to the satisfaction questionnaires, [36] and the doctor’s confidence in a certain time. Furthermore, we collected other information from all trainees in both groups, including participation in the “Star Family Doctors Training program for the management of chronic obstructive pulmonary disease,” which was also sponsored by the Health Commission of Sichuan Province, and the academic participation of medical association.

### Quality control

The training contents were designed by the teaching team of the Department of General Practice of West China Hospital based on the preliminary research results. All trainers were doctors from the departments of general practice, cardiology, endocrinology, and respiratory and critical care medicine of West China Hospital. All trainers had a college teacher qualification certificate and a teaching certificate for the standardized training of residents. The trainers and training contents for both the STAR group and the control group were identical. There was no learning interaction between trainees from different groups during the program.

### Data analysis

Statistical analysis was performed using SPSS software (version 12.0; SPSS Inc.). Continuous data were expressed as the mean ± standard deviation, while discrete data were expressed as the number and ratio. The chi-squared test was used to compare categorical variables. The independent or paired *t*-test was used to compare mean values between different groups. A probability (P) value of < 0.05 was considered statistically significant.

## Results

### Demographic characteristics of the participants

A total of 80 GPs from the PHC system in Sichuan Province were enrolled in this study. The baseline demographic information and comparison of the participants in the two study groups are shown in Table 4. The proportion of participants with intermediate or senior professional titles was higher in the STAR group than in the control group (*p* = 0.002). The participants in the STAR group had more years of work experience than those in the control group (*p* = 0.01). Furthermore, the proportion of township health care center doctors was significantly higher in the STAR group than in the control group (*p* = 0.02).

**Table 4:** Demographic descriptions of both the STAR group and the control group

		Groups			
Characteristics	No. (n = 80)	STAR (n = 30)	Control (n = 50)	t/χ^2^ value	*P* values
Age (years)	80 (100%)	39.47 ± 6.61	36.34 ± 5.41	1.17	0.25
Gender					
Male	23 (28.75%)	11 (36.7%)	12 (24%)	1.47	0.23
Female	57 (71.25%)	19 (63.3%)	38 (76%)		
Practicing as GP (years)	80 (100%)	16.57 ± 7.08	12.78 ± 5.81	2.60	**0.01**
Professional titles					
Primary	21 (26.25%)	2 (6.7%)	19 (38%)	11.74	**0.002**
Intermediate	50 (62.5%)	22 (73.3%)	28 (56%)		
Senior	9 (11.25%)	6 (20%)	3 (6%)		
Type of healthcare institution					
Private clinics	4 (5%)	0	4 (8%)	7.01	**0.02**
Urban primary healthcare centers	54 (67.5%)	17 (56.7%)	37 (74%)		
Township healthcare centers	22 (27.5%)	13 (43.3%)	9 (18%)		

*Abbreviations*: STAR = Star Family Doctors Training Program; GP = General practitioners.

### Immediate post-training evaluation

#### Post-training tests

**Fig. 1:**
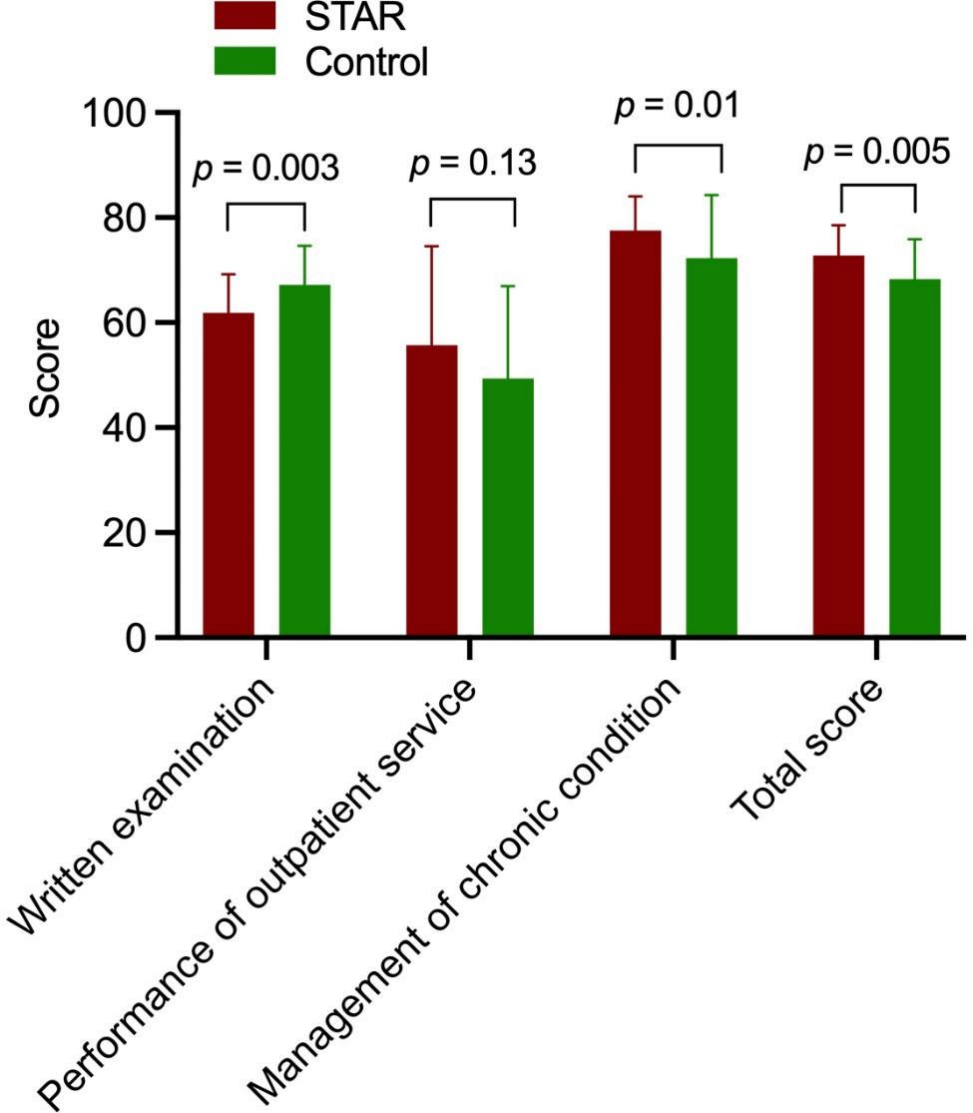
The comparison of post-training test results of both groups. Score of the performance of outpatient service including physical examination assessment and inquiry assessment. Score of the management of chronic condition including SOAP medical record writing and presentation of chronic condition management

All 30 trainees in the STAR group and 47 of the 50 trainees in the control group acquired the CME certification, including 5 trainees in the STAR group and 3 trainees in the control group who were appraised as “excellent” based on their total scores. The average post-training total score was significantly higher in the STAR group than in the control group (72.83 ± 5.73 vs. 68.18 ± 7.64; *p* = 0.005; Fig. 1). In addition, the presentation of chronic-condition management was better in the STAR group than in the control group (*p* = 0.01). In contrast, the average written examination score was significantly higher in the control group than in the STAR group (*p* = 0.003).

### Trainees’ self-evaluation of training courses and tests

The top 10 courses that all trainees from both groups considered they benefitted the most from were as follows: dyslipidemia and atherosclerosis (n = 67, 83.8%), physical examination (n = 65, 81.3%), common electrocardiogram abnormality identification (n = 61, 76.3%), common chest imaging abnormality identification (n = 61, 76.3%), common laboratory test abnormality identification (n = 61, 76.3%), hypertension (n = 56, 70%), diabetes mellitus (n = 56, 70%), chronic pulmonary obstructive disease (n = 55, 68.8%), hyperuricemia and gout (n = 55, 68.8%), and whole-person health management experience sharing (n = 51, 63.8%). In terms of the assessments, most trainees considered that they benefitted the most from the following: presentation of chronic-condition management (n = 63, 78.8%), physical examination assessment (n = 45, 56.3%), and inquiry assessment (n = 42, 52.5%).

Table 5 shows a comparison of the trainees’ immediate post-training self-evaluation of the training courses and design between the two groups based on 5-point Likert scale. Compared to control group, most trainees in the STAR group highly regarded the enhancement of clinical ability (80% vs. 42%, *p* = 0.001), tightness of the training to the practical needs of community (70% vs. 38%, *p* = 0.006), assistance to the standardization of diagnosis and treatment (90% vs. 52%, *p* = 0.001), improvement of teaching ability (76.7% vs. 32%, *p* = 0.001), and inclination of recommending colleagues to similar training (90% vs. 64%, *p* = 0.01), respectively.

**Table 5:** Five-degree Likert scale of post-training trainees’ evaluation of the training program

		Groups			
Characteristics	No. (n = 80)	STAR (n = 30)	Control (n = 50)	χ [2] value	*P* values
Likert 5-degree scale grading at 5 points					
Enhancement of clinical ability	45 (56.25%)	24 (80%)	21 (42%)	11.0	**0.001**
Tightness of the training to the practical needs of community	40 (50%)	21 (70%)	19 (38%)	7.68	**0.006**
Accuracy and the key-points emphasis of the training	36 (45%)	17 (56.7%)	19 (38%)	2.64	0.10
Assistance to the standardization of diagnosis and treatment	53 (66.25%)	27 (90%)	26 (52%)	12.11	**0.001**
Improvement of teaching ability	39 (48.75%)	23 (76.7%)	16 (32%)	14.97	**0.001**
Inclination of recommending colleagues to similar training	59 (73.75%)	27 (90%)	32 (64%)	6.55	**0.01**

*Abbreviations*: STAR = Star Family Doctors Training Program.

### The 12-month follow-up

According to the results of the follow-up survey, STAR participants reported seeing more patients (all *p* < 0.05), and had more patients who signed family-doctor contracts (*p* = 0.001), as well as increased patient satisfaction (*p* = 0.03), respectively, (Table 6). At the 12-month follow-up, the majority of the trainees, regardless of study group, (n = 65, 81.3%) reported that they frequently made full use of the training contents in their daily clinical practice. In addition, more than half of all trainees (n = 51, 63.8%) considered themselves to be “obviously more confident in daily work with patients” after having completed the training program.

**Table 6:** Summary of the 12-month follow-up of both groups

		Groups			
Characteristics	No. (n = 80)	STAR (n = 30)	Control (n = 50)	χ [2] value	*P* values
Practical application of the training contents					
Frequently	65 (81.25%)	28 (93.3%)	37 (74%)	4.60	**0.03**
Occasionally	15 (18.75%)	2 (6.7%)	13 (26%)		
Hardly	0	0	0		
The way of promotion of the training contents					
Through public lecture	31 (38.75%)	19 (63.3%)	12 (24%)	14.95	**0.001**
Through daily communication	46 (57.5%)	9 (30%)	37 (74%)		
Through administrative means	3 (3.75%)	2 (6.7%)	1 (2%)		
Changes of the numbers of the regular outpatients in a certain time					
No	18 (22.5%)	2 (6.7%)	16 (32%)	7.81	**0.02**
Elevated by 10-30%	57 (71.3%)	25 (83.3%)	32 (64%)		
Elevated by 30-50%	5 (6.3%)	3 (10%)	2 (4%)		
Elevated by more than 50%	0	0	0		
Changes of the numbers of the contracted patients of the family doctor signing service in a certain time					
No	30 (37.5%)	4 (13.3%)	26 (52%)	13.94	**0.001**
Elevated by 10-30%	46 (57.5%)	24 (80%)	22 (44%)		
Elevated by 30-50%	3 (3.8%)	1 (3.3%)	2 (4%)		
Elevated by more than 50%	1 (1.3%)	1 (3.3%)	0		
Changes of the patients’ satisfaction in a certain time					
No	13 (16.3%)	1 (3.3%)	12 (24%)	5.81	**0.03**
Elevated	67 (83.8%)	29 (96.7%)	38 (76%)		
Changes of the doctor’s confidence in a certain time					
No	2 (2.5%)	0	2 (4%)	5.43	**0.05**
Slightly	27 (33.8%)	6 (20%)	21 (42%)		
Obviously	51 (63.8%)	24 (80%)	27 (54%)		

*Abbreviations*: STAR = Star Family Doctors Training Program.

## Discussion

Since 2000, the Chinese government has sought curriculum assistance from the World Organization of Family Doctors to improve the Chinese education, training, and practice system. Among these system, CME is a very important aspect, but unlike the developed country, there is not a complete CME system for rural healthcare workers in China such as Sichuan Province. Therefore, the government of China has aimed to improve the general practice literacy of rural healthcare workers through various CME programs. In this study, the STAR presented new ideas for the training of GPs in rural area in China through an innovative CME model. The program yielded good outcomes and provided a good reference point for similar programs in the future.

Primary health care emphasized first-contact, accessible, continued, comprehensive, and coordinated patient-focused care, and is often the closest to where people live. Primary health care has been recognized as the cornerstone of combating non-communicable diseases. In China, the primary health care system is comprised of three tiers of healthcare providers: village clinics, township health centers, and county hospitals. Under China’s guidelines for non-communicable diseases management within the rural healthcare system, primary care providers from village clinics and township health centers take primary responsibility in diagnosing patients during their initial visits and treating the diagnosed patients. First-time patients are recommended to visit a village clinics and township health centers, and to then visit a county hospitals if their health issues remain unresolved. [14].

The STAR Family Doctors Training Program, as part of the provincial primary care system quality promotion projects strongly supported by the Health Commission of Sichuan Province, offers multiple advantages over many previously reported CME programs. [25–32] First, the local health administration department takes the lead in implementing the program; thus, GPs from all PHC institutions across Sichuan Province, including distant or less-developed regions, have the chance to participate in this program. Our findings show that the proportion of GPs from township health care centers was significantly higher in the STAR group than in the control group. Moreover, 70% of the trainees in the STAR group considered the training contents to have a very strong or strong correlation with the practical needs of the community, while this proportion was only 38% in the control group. Furthermore, 90% of the trainees in the STAR group strongly agreed or agreed to recommend similar training to their colleagues, which was significantly higher than the corresponding proportion in the control group. Consistent with results in our study, some study also reported that They reported a strong need for CME to upgrade their knowledge and skills because they attained junior college education or blow in China. [37] These findings prove that the STAR Family Doctors Training Program possesses a wider coverage of GPs from distant and medically underserved areas, and is more valued by those trainees.

Second, participants of the STAR group were selected from their own institutions, and many of them worked as the heads of their departments/centers or held administrative positions; therefore, it was easier for them to apply or promote the training contents to both their patients and colleagues. Approximately 77% of the trainees in the STAR group stated that they “strongly agreed or agreed with the statement that their teaching ability had improved due to the program,” which was significantly higher than the corresponding proportion in the control group. In addition, the majority of the trainees in the STAR group considered that they “frequently apply the training contents during daily work,” and this proportion was also significantly higher than that in the control group. Finally, 63.3% of the trainees in the STAR group and only 24% of the trainees in the control group promoted the training contents through public lectures. These findings indicate that the preliminary selection of the trainees by their own institutions may improve the promotion and application of the training.

The STAR Family Doctors Training Program was designed as an integrated small group learning program with plenty of time for group discussions and workshop practice, the use of standard patients and medical patient simulators, and an emphasis on post-training assessment. The application of multiple teaching methods and the conventional face-to-face training design provided trainees from PHC institutions with valuable opportunities to practice on equipment, express themselves, share work experiences, and solve learning difficulties with each other. In recent years, remote education has become the trend in CME, [21] yet some studies show that many GPs remain interested in attending face-to-face training sessions with colleagues.[28, 38−39] Considering that most of the participants from PHC institutions had not undergone standard patient training when they had attended medical school, we specifically used standard patients from West China Hospital in our physical examination and inquiry training sessions. Previous CME programs have usually adopted written examinations and self-reporting for the short-term evaluation of the training results. [26, 28] It is worth noting that our short-term evaluation showed that the average written examination score was significantly higher in the control group than in the STAR group. In contrast, the average total score, including the scores of the physical examination assessment, inquiry assessment, SOAP medical record writing, and presentation of chronic-condition management, was significantly higher in the STAR group than in the control group. These results indicate that adopting written examination as the main evaluation method may not be a good way of promoting and sustaining practical clinical ability. Multiple assessment methods, including the presentation and sharing of a real-world case, and inquiry and physical examination assessment using a standard patient, could better improve the comprehensive ability of the trainees.

At the 12-month follow-up assessment, we found that 63.3% of the trainees in the STAR group chose to share or promote the training contents/experience with their colleagues through public lectures, which was significantly higher than the corresponding proportion in the control group. This may be because more trainees in the STAR group held higher administrative positions or worked as the headers of their units, thus making it easier for them to promote what they had learned. In addition, the trainees in the STAR group acquired more patients under a family-doctor contract from the family-doctor signing service within 1 year after the training, as compared with the control group. The patients of the STAR-group trainees were more satisfied with the medical services they received than the patients of the control-group trainees. This may be because the trainees from the STAR group obtained certificates authorized by the Health Commission of Sichuan Province; therefore, they may have gained the trust of their patients. Moreover, the special “Bidirectional Referral Medical Service of General Practice Community Alliance of West China Hospital” helped to ensure that patients could be referred to the Department of General Practice of West China Hospital when the trainees encountered difficult or severe medical situations, thus increasing their patients’ willingness to sign a family-doctor contract with these GPs. This novel training setting, which combines CME with authorized certification and two-way referral mechanisms, has not previously been reported. In addition, this new training model has shown good long-term effects in terms of practical application of the training contents. It is hoped that through the conclusion of this study, the health administration department will support the CME of grassroots doctors in more aspects in the future, not only in terms of funding but also in terms of qualification certification and medical service management.

Generally, trainees in the STAR group appraised the training program setting higher than did the trainees in the control group. Among all the training courses, physical examination, electrocardiogram abnormality identification, chest imaging abnormality identification, and laboratory test abnormality identification were listed as the top ranked courses favored by trainees from both groups. In terms of the assessments, most trainees considered that they benefitted the most from the assessment of the presentation of chronic-condition management, followed by physical examination assessment and inquiry assessment. Previous CME studies for PHC GPs have focused on the management of specific diseases instead of on basic clinical medical skills, such as electrocardiography, laboratory testing, medical imaging, and physical examination. [26–30] However, based on the feedback of the trainees, future training design should emphasize practical clinical skills suitable for GPs. Additionally, more emphasis should be placed on practical application and processing than on theoretical principles.

According to the trainees’ evaluation of the training program setting, more members of the STAR group than of the control group believed that the training enhanced their clinical-practice ability and their teaching ability. It is worth noting that the training course contents of the STAR group were completely consistent with those of the control group, and there was no teaching-related curriculum design. Thus, the above findings may be attributable to differences in the delivery of the training courses between the two groups. In addition to lectures and workshops, methods such as standard patients, small group case studies, and medical patient simulators were used in the STAR group; this provided more opportunities for the trainees to practice and discuss with each other and therefore acquire some teaching methods during the training.

This study has several strengths. This study is one of a few cohort studies to investigate the effectiveness of a comprehensive CME program designed to enhance GPs’ practice, knowledge, and confidence in the management of common chronic diseases as well as community-based medical technology in general practice. The delivery and evaluation of this program involved collaboration with “West China Hospital and Chenghua Urban Area Medical Service Alliance” and the Health Commission of Sichuan Province. It synthesized data from comprehensive objective assessments, trainees’ self-evaluations, program evaluations, and process evaluations to identify current gaps in GPs’ awareness, track the effectiveness of the program in improving self-reported capacity, and identify areas for future improvements.

However, further study will provide comprehensive insight into root causes of current challenges by exploring the evolution and socioeconomic environment of primary care system on a large scale, such as lack opportunity, heavy workload, lack of time, the high cost of courses, reformed face-to-face training skill, and lack of organizational coordination, that hindered GPs from participating CME, especially rural area in China. Based on previous studies, it is feasible to conduct large scale E-learning CME activities for populous countries such as China, Brazil, and India facing similar challenges. [40] Compared with traditional face-to-face learning, several studies shown that physicians favored the E-learning’s unprecedented value in knowledge transmission and accessibility. [41] Based on previous studies, face-to-face training is more suitable for operational courses such as physical examination, which not only allows students to have hands-on opportunities, however, for theoretical courses, the advantages of E-learning became apparent. Therefore, E-learning and traditional face-to-face learning are complementary, and the blended learning of face-to-face learning and E-learning in CMEs, such as blended learning is a question for future studies. [42].

The limitations of this study include a relatively small sample size, the use of a purpose-designed survey, the lack of pretest assessment data, and the use of self-reported data in the follow-up assessment. Considering that the research was designed as a combination of small group discussions with plenty of time for practice, it was relatively difficult to include a large number of participants. While participant self-selection in the control group could have introduced bias, one of the purposes of the study was to demonstrate the advantages and disadvantages of self-selection vs. the recommended selection of participants. We did not employ a pretraining test in our study because it is possible that completing the test prior to engaging in the CME program could have raised the participants’ awareness of specific topics and therefore altered how they approached the CME program as well as their responses on the post-training tests.

## Conclusion

In conclusion, the Star Family Doctors Training Program presents new ideas for the training of GPs in China through an innovative CME model. The program yielded good outcomes and provided a good reference point for similar programs in the future.

## Electronic supplementary material

Below is the link to the electronic supplementary material.

**Supplementary file 1**. Teaching contents and methods of the control group

## Data Availability

The datasets used and/or analysed during the current study available from the corresponding author on reasonable request.
