# Supplementary material for: Evaluation of the star family doctors training program: an observational cohort study of a novel continuing medical education program for general practitioners within a compact medical consortium: a quantitative analysis
Source: BMC Med Educ. 2023 Apr 17;23:250. doi: 10.1186/s12909-023-04210-7 (PMC10108467; doi:10.1186/s12909-023-04210-7)
Supplement: Supplementary file 1 — Teaching contents and methods of the control group [file 12909_2023_4210_MOESM1_ESM.docx]

**Supplementary file 1**. Teaching contents and methods of the control group

| Items | Courses | Methods | Time allocation |
| --- | --- | --- | --- |
| *Fundamental GP clinical skills* | Consultation skills and doctor-patient communication | Lectures | 1 hour |
|  | SOAP medical record writing | Lectures | 1 hour |
|  | Physical examination: including (1) Head and Neck; (2) Chest; (3) Abdomen; (4) Extremities. | Lectures and demonstration | 2.5 hours for each part |
| *Fundamental conceptions of general practice* | 1. Clinical thinking of general practitioners 2. Whole-person health management experience sharing 3. Design and implementation of the contracted health service package 4. Operational management of the health management team | Lectures and case sharing | 2 hours  2 hours  2 hours  2 hours |
| *Requisite skills for daily clinical practice in the community* | 1. Portable pulmonary function devices 2. Ambulatory blood pressure monitor 3. Dynamic blood glucose monitor 4. Patient education | Lectures, demonstration, and workshop practice | 2.5 hours  2.5 hours  2.5 hours  2 hours |
| *Requisite abilities for daily clinical practice in the community* | 1. Cardio-pulmonary resuscitation and the Heimlich maneuvere 2. Thoracentesis, abdominocentesis, debridement, wound treatment 3. Common Electrocardiogram abnormality identification 4. Common chest imaging abnormality identification 5. Common Color Doppler ultrasound abnormality identification 6. Common laboratory tests abnormality identification | Lectures, demonstration and image/ECG reading practice | 2 hours  2 hours  1.5 hours  1.5 hours  1.5 hours  1.5 hours |
| *Holistic common chronic condition management* | (1) Hypertension; (2) Diabetes mellitus; (3) Dyslipidemia and atherosclerosis; (4) Chronic pulmonary obstructive disease; (5) Hyperhyperuricemia and gout; (6) coronary heart disease; (7) Chronic heart failure; (8) Ischemic stroke and intracerebral hemorrhage; (9) Osteoporosis; (10) Prostatomegaly. | Lectures | 2 hours for each session |
